# Preferences for attributes of oral antipsychotic treatments: results from a discrete-choice experiment in respondents with schizophrenia or bipolar I disorder

**DOI:** 10.1186/s12888-024-06034-1

**Published:** 2024-09-10

**Authors:** Michael J. Doane, Marco Boeri, Caroline Vass, Cooper Bussberg, Hemangi R. Panchmatia, Leslie Citrome, Martha Sajatovic

**Affiliations:** 1 Health Economics and Outcomes Research, Alkermes, Inc., 900 Winter St., Waltham, MA 02451-1420 USA; 2 Health Preference Assessment, RTI Health Solutions, Belfast, Northern Ireland, UK; 3 Queen’s University Belfast, Belfast, Northern Ireland, UK; 4 University of Manchester, Manchester, UK; 5 Health Preference Assessment, RTI Health Solutions, Manchester, UK; 6 Health Preference Assessment, RTI Health Solutions, Research Triangle Park, NC USA; 7 New York Medical College, Valhalla, NY USA; 8 University Hospitals Cleveland Medical Center, Case Western Reserve University School of Medicine, Cleveland, OH USA

**Keywords:** Patient preference, Treatment efficacy, Weight gain, Sedation, Discrete-choice experiment

## Abstract

**Background:**

Antipsychotic medications are effective treatments for schizophrenia (SZ) and bipolar I disorder (BD-I), but when presented with different treatment options, there are tradeoffs that individuals make between clinical improvement and adverse effects. As new options become available, understanding the attributes of antipsychotic medications that are valued and the tradeoffs that individuals consider when choosing among them is important.

**Methods:**

A discrete-choice experiment (DCE) was administered online to elicit preferences across 5 attributes of oral antipsychotics: treatment efficacy (i.e., improvement in symptom severity), weight gain over 6 months, sexual dysfunction, sedation, and akathisia. Eligible respondents were aged 18–64 years with a self-reported clinician diagnosis of SZ or BD-I.

**Results:**

In total, 144 respondents with SZ and 152 with BD-I completed the DCE. Of those with SZ, 50% identified themselves as female and 69.4% as White, with a mean (SD) age of 41.0 (10.1) years. Of those with BD-I, most identified themselves as female (69.7%) and as White (77.6%), with a mean (SD) age of 40.0 (10.7) years. In both cohorts, respondents preferred oral antipsychotics with better efficacy, less weight gain, no sexual dysfunction or akathisia, and lower risk of sedation. Treatment efficacy was the most important attribute, with a conditional relative importance (CRI) of 31.4% for respondents with SZ and 31.0% for those with BD-I. Weight gain (CRI = 21.3% and 23.1%, respectively) and sexual dysfunction (CRI = 23.4% and 19.2%, respectively) were adverse effects in this study that respondents most wanted to avoid. Respondents with SZ were willing to accept 9.8 lb of weight gain or > 25% risk of sedation for symptom improvement; those with BD-I were willing to accept 8.5 lb of weight gain or a > 25% risk of sedation.

**Conclusions:**

In this DCE, treatment efficacy was the most important attribute of oral antipsychotic medications among respondents with SZ and BD-I. Weight gain and sexual dysfunction were the adverse effects respondents most wanted to avoid; however, both cohorts were willing to accept some weight gain or sedation to obtain better efficacy. These results highlight features that patients value in antipsychotic medications and how they balance benefits and risks when choosing among treatments.

**Supplementary Information:**

The online version contains supplementary material available at 10.1186/s12888-024-06034-1.

## Background

Antipsychotic medications are effective in managing the symptoms of schizophrenia (SZ) and bipolar I disorder (BD-I) [1, 2]. Despite their clinical utility, these agents can be associated with adverse effects such as weight gain, sedation, sexual dysfunction, and movement disorders [1, 3]. Individuals with SZ or BD-I who experience these adverse effects often find them bothersome and ultimately may choose to discontinue treatment because of them [4, 5]. As new medication options become available, there is a need to better understand the features of antipsychotic medications that patients value, along with the tradeoffs that may be acceptable when choosing among them. By considering these tradeoffs and addressing patient concerns, clinicians may be able to tailor treatment options that ultimately improve medication adherence and patient outcomes.

Stated-preference methods are survey-based approaches for evaluating preferences for healthcare outcomes, products, and services [6, 7]. As they relate to this study, discrete-choice experiments (DCEs) use a stated-preference method in which respondents are presented with profiles of hypothetical treatments that vary across different levels (e.g., degree of medication efficacy or amount of weight gain). From choices made over many scenarios, it is possible to determine how respondents balance different product attributes and evaluate the relative importance of each attribute [8].

Several studies have employed a stated-preference methodology in an effort to understand the attributes that individuals living with SZ or BD-I and their clinicians value in antipsychotic medications [9–11]. These studies have usually included attributes associated with clinical or functional treatment efficacy (e.g., symptom improvement, social functioning), as well as adverse effects (e.g., weight gain, sedation, extrapyramidal symptoms).

Historically, improvement of symptoms (efficacy) has been considered the most important attribute of a hypothetical antipsychotic medication [9, 11, 12]. With respect to adverse effects, weight gain and adverse metabolic effects were identified as the most important to avoid [9–13]. However, research on preferred antipsychotic medication attributes has typically focused on the relative importance of individual attributes but has paid less attention to the tradeoffs that individuals may be willing to make to achieve a favorable balance of benefit and risk.

In this study, we used a DCE to assess preferences of individuals with SZ or BD-I for attributes associated with oral antipsychotic medications and further explored potential tradeoffs that they may make between efficacy and adverse effects in their choice of antipsychotic medication.

## Methods

### Study design

This noninterventional, cross-sectional study included a DCE survey instrument to elicit preferences for different attributes of oral antipsychotic medications [14, 15]. The DCE methodology is based on the principle that treatments are characterized by various attributes and that a respondent’s choice of treatment is determined by their utility gain with one alternative compared with another, which is a function of the utility of each attribute considered, and of the respondent’s preferences [7]. DCEs are commonly used to elicit tradeoffs that respondents make among multiple treatment attributes.

The DCE survey instrument queried respondents to respond to a series of choices between pairs of hypothetical oral antipsychotic medications. The DCE was designed in accordance with good research practices as outlined by the Professional Society for Health Economics and Outcomes Research (ISPOR) guidelines [6]. All procedures were carried out in accordance with the Declaration of Helsinki and were approved by the institutional review board of RTI International.

### Development of the DCE

The antipsychotic medication attributes included in the DCE survey instrument were selected based on a systematic literature review of attribute-based stated-preference studies in individuals with schizophrenia, schizoaffective disorder, or bipolar disorder (Additional File 1). PubMed and Medline were searched for relevant records of research articles (Additional File 2) from January 1, 1990, to April 23, 2021; additional records of abstracts were obtained via the ISPOR and American Psychiatric Association websites. Records were then screened independently by M.B. and C.V. for eligibility and to identify and remove duplicates. The final literature review included 17 articles pertaining to preferences for antipsychotic medications in individuals with schizophrenia, schizoaffective disorder, or bipolar disorder.

Based on the results of this review and patient feedback obtained in previous studies, the following 5 key attributes were selected: treatment efficacy (i.e., improvement in symptom severity), weight gain over 6 months, sexual dysfunction, sedation, and akathisia. Each attribute was further characterized by 2 to 4 corresponding levels (Table 1).

**Table 1:** Treatment attributes

Attribute	Level
Treatment efficacy (improvement in symptom severity)	A lot of improvement (from severe to no symptoms)
	Some improvement (from severe to mild symptoms)
	A little improvement (from severe to moderate symptoms)
Weight gain	No weight gain
	4-lb weight gain
	7-lb weight gain
	11-lb weight gain
Sexual dysfunction	No
	Yes
Treatment-related akathisia	No
	Yes
Risk of sedation	None
	10 of 100 people (10%)
	25 of 100 people (25%)

For the treatment efficacy attribute, levels were derived from psychometric evaluations of psychiatric illness severity in patients with SZ or BD-I [16, 17]. The corresponding treatment efficacy level options included “a lot of improvement (from severe to no symptoms),” “some improvement (from severe to mild symptoms),” and “a little improvement (from severe to moderate symptoms).” For the weight gain attribute, a previous clinical trial on the potential for gaining weight during treatment with olanzapine informed the levels chosen [18]. The corresponding levels were no weight gain, 4 lb of weight gain, 7 lb of weight gain, and 11 lb of weight gain. The attributes of sexual dysfunction, akathisia, and sedation were selected based on reports that they are common and bothersome adverse effects of antipsychotic medications [4, 5, 19]. The sexual dysfunction and akathisia attributes were assessed using “no” or “yes” responses. The risk of sedation attribute was assessed using the responses “none,” “occurs in 10 of 100 people (10%),” and “occurs in 25 of 100 people (25%).”

Before implementation of the full online survey, a draft DCE was pretested in adults with SZ (*n* = 15) or BD-I (*n* = 15) from March 14, 2022, through April 8, 2022, to ensure its comprehension and to assess the cognitive burden that it imposed. Eligible participants were US residents aged 18 to 64 years with a self-reported diagnosis of SZ or BD-I who had access to the internet. Participants were excluded if they had been hospitalized for psychosis within 3 months before completing the DCE or if they had a conservator, trustee, or legal guardian making decisions on their behalf. Each participant provided written informed consent. The informed consent form explained that participants would be asked to take a survey to help us understand treatment preferences of people living with a serious mental health disorder.

During the pretest, participants reviewed choice questions with multiple variations, and this feedback was used to refine the final DCE. Participants engaged in cognitive qualitative interviews and verbalized their answers as they completed the draft DCE. After completion, pretest participants were asked a series of debriefing questions to determine whether they understood the survey instructions and medication attributes. Participants identified any text that was confusing or incorrect, as well as any information of interest to them that was omitted or not described in sufficient detail. Based on this feedback, the descriptions of the attributes and levels were simplified, and the number of levels was reduced where possible to reduce cognitive burden. Data collected during the pretest interviews were not included in the preference analyses reported below.

**Fig. 1:**
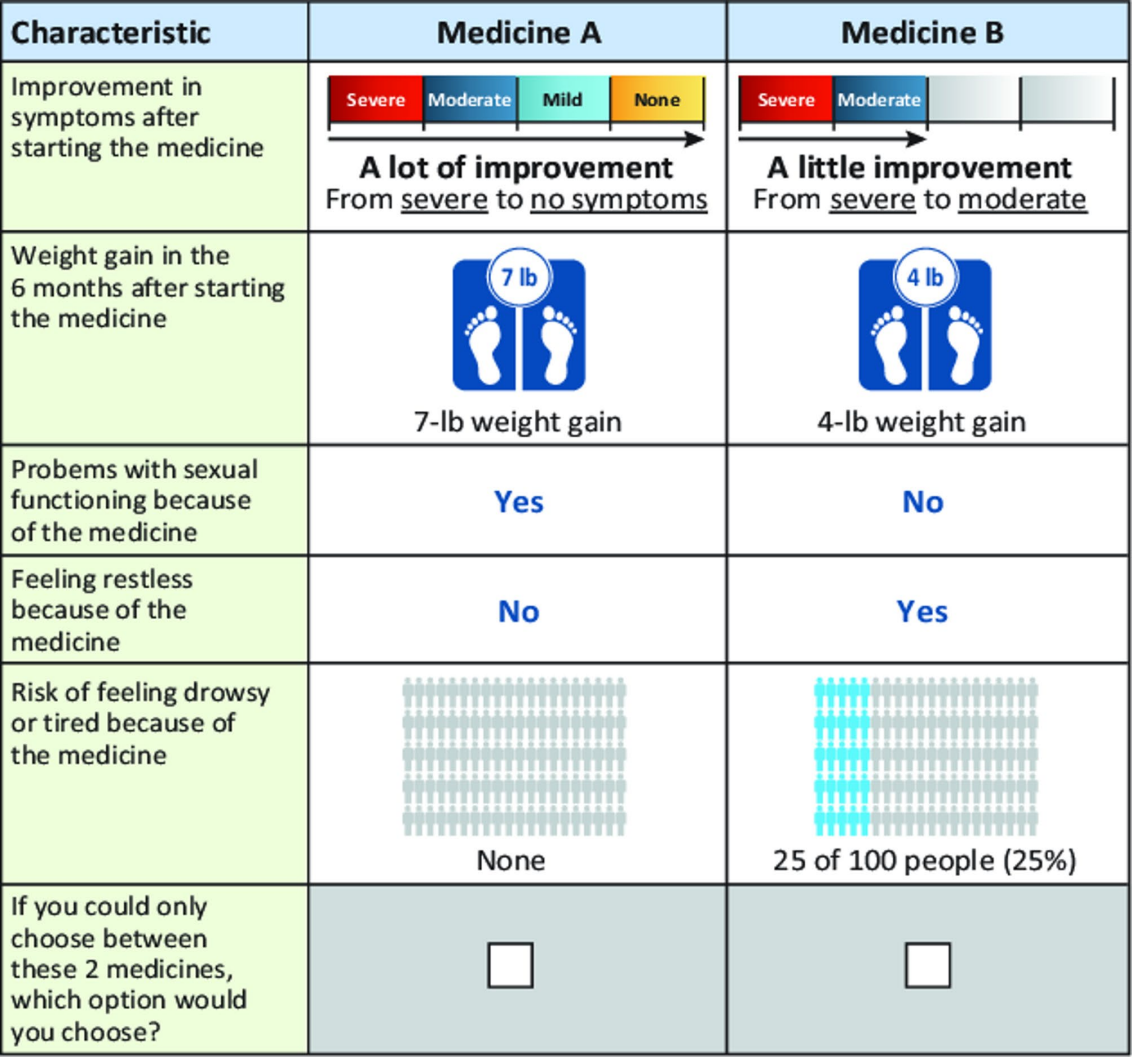
Example choice set

The combination of levels used to define each treatment profile, the set of profiles in each choice question (Fig. 1), and the full set of choice questions in a DCE is known as the experimental design. The fractional factorial experimental design was constructed using a D-optimal algorithm [20]. The design was statistically efficient, thus isolating the effects of individual attributes and ensuring sufficient variation across choice sets. These design properties helped to mitigate the potentially confounding effects of multiple attribute changes.

The final survey contained 72 DCE questions that were used to create 6 blocks of 12 DCE questions each. Respondents were randomly assigned to 1 block of 12 questions, and the questions were ordered randomly within each block to avoid ordering effects. In addition, screening, demographic, and practice questions were included.

For each question in the DCE, 2 hypothetical antipsychotic medications with different attribute levels were presented. For each attribute, 1 or 2 questions designed to encourage participants to think about the attribute, break up the text describing the attributes, and assess the participants’ comprehension of the attribute were asked. Participants then chose their preferred option.

The final survey instrument was programmed and administered online between June 21, 2022, and September 26, 2022, to participants who were identified using patient databases and organizations, clinician referrals, and social media advertising. Eligible participants were US residents meeting the same inclusion and exclusion criteria noted above for the pretest DCE interviews. Each participant received a unique link to the DCE survey via email.

No formal power analysis was conducted because a priori effect sizes were unknown. Researchers employing stated-preference methodology often use a minimum of 150 respondents per group [21]; therefore, sample sizes of 150 respondents with SZ and 150 with BD-I were considered sufficient for analysis. All participants provided informed consent. The survey instrument questions can be found in Additional File 3.

### Statistical analysis

Demographic and clinical characteristics were summarized descriptively by cohort. Preference data were analyzed using random-parameter logit (RPL) models.

The DCE analysis produced preference coefficient estimates for the full set of attribute levels. The conditional relative importance (CRI) of each attribute was calculated as the difference between the preference coefficient estimate of the least-preferred and most-preferred levels. The CRI estimates were rescaled such that their sum was equal to 100 and can be interpreted as the proportion of utility gained by improving each attribute from the least- to the most-preferred level relative to the maximum utility that can be gained from improving all attributes. The delta method was used to compute 95% CIs [22].

Preference coefficient estimates were used to calculate the maximum acceptable weight gain (MAWG) and maximum acceptable risk (MAR) of sedation that respondents were willing to accept for a given increase in treatment efficacy or other treatment benefit(s). The MAWG was defined as the negative ratio between the marginal utility of a specific improvement in an attribute and the marginal disutility of 1 lb of weight gain. Given the following hypothetical preference coefficient estimates,

- utility of going from “a little” to “some” disease improvement, 1.5, and
- disutility of an increase in weight gain from 0 to 4 lb, − 0.5,

the MAWG was calculated as follows:

$$\:\text{M}\text{A}\text{W}\text{G}\:=\:-\frac{1.5}{\frac{-0.5}{4\:-\:0}}=\:12\:\text{l}\text{b}$$

The MAR was defined as the negative of the ratio between the marginal utility of a specific improvement in an attribute and the marginal disutility of each risk. Given the following hypothetical preference coefficient estimates,

- utility of going from “a little” to “some” disease improvement, 1.5, and
- disutility of an increase in the risk of sedation from 0 to 10%, − 0.5,

the MAR was calculated as follows:

$$\:\text{M}\text{A}\text{R}\:=\:-\frac{1.5}{\frac{-0.5}{10\:-\:0}}=\:30\%$$

Details of the RPL models and the full equations for the MAWG and MAR can be found in Additional File 4.

All analyses were conducted using Stata 17 (College Station, TX, USA).

## Results

### Demographic and clinical characteristics

Overall, 1837 potential respondents accessed the survey. Of these, 1541 were excluded for the following reasons: not meeting the screening criteria (*n* = 1247), exceeding the number of respondents with BD-I needed for analysis (*n* = 264), providing an incomplete survey (*n* = 29), or having no variability in their answers (*n* = 1).

A total of 144 respondents with SZ and 152 with BD-I completed the DCE (Table 2). The median time to complete the survey was 20.8 min. Of those respondents with SZ, 50.0% identified themselves as female and 69.4% as White. The mean (SD) age was 41.0 (10.1) years, and the mean (SD) age at diagnosis was 30.0 (10.5) years. Of the respondents with BD-I, most identified as female (69.7%) and as White (77.6%), with a mean (SD) age of 40.0 (10.7) years. The mean (SD) age at diagnosis for BD-I respondents was 25.0 (9.1) years.

**Table 2:** Demographic characteristics

Parameter	SZ Cohort (*n* = 144)	BD-I Cohort (*n* = 152)
Sex, female, *n* (%)	72 (50.0)	106 (69.7)
White, *n* (%)	100 (69.4)	118 (77.6)
Age, mean (SD), years	41.0 (10.1)	40.0 (10.7)
Age at diagnosis, mean (SD), years	30.4 (10.5)	25.4 (9.1)
Time since diagnosis, mean (SD), years	10.6 (9.7)	15.0 (10.2)
≤ 5 years since diagnosis, *n* (%)	64 (44.4)	34 (22.4)
Oral antipsychotic treatment exposure		
FGA, *n* (%)^a^		
Currently taking	34 (23.6)	9 (5.9)
Taken in the past	64 (44.4)	47 (30.9)
SGA, *n* (%)^a^		
Currently taking	91 (63.2)	59 (38.8)
Taken in the past	71 (49.3)	97 (63.8)
Symptom severity in past week, *n* (%)		
No symptoms	6 (4.2)	14 (9.2)
Mild	34 (23.6)	35 (23.0)
Moderate	65 (45.1)	57 (37.5)
Severe	39 (27.1)	46 (30.3)
BMI, mean (SD), kg/m^2^	32.0 (8.6)	32.6 (9.1)
BMI category, *n* (%)		
Overweight (BMI > 25.0 to < 29.9 kg/m^2^	43 (29.9)	30 (19.7)
Obese (BMI ≥ 30.0 kg/m^2^)	75 (52.1)	84 (55.3)
Experienced antipsychotic adverse effects, *n* (%)		
Weight gain	123 (85.4)	126 (82.9)
Drowsiness	118 (81.9)	142 (93.4)
Restlessness	102 (70.8)	110 (72.4)
Sexual dysfunction	92 (63.9)	105 (69.1)

^a^Respondents could select multiple responses; therefore, totals may not equal the number of respondents

BD-I, bipolar I disorder; BMI, body mass index; FGA, first-generation antipsychotic; SGA, second-generation antipsychotic; SZ, schizophrenia

### Preference analysis

#### Schizophrenia

**Fig. 2:**
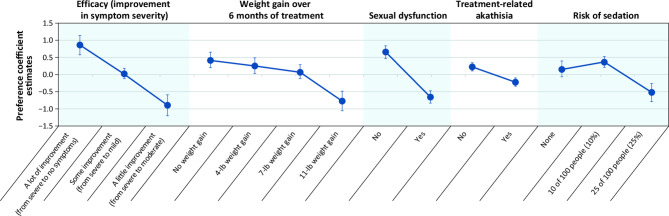
Preferences for attributes of oral antipsychotic medications for respondents with schizophrenia^**a**^ ^a^Preference coefficient estimates are presented along with their 95% confidence intervals, with higher estimates for a given level associated with a greater preference for that level. The vertical distance between any 2 levels of an attribute represents the change in utility; larger differences indicate that respondents viewed the change as having a relatively greater effect on overall utility. Attributes are presented in the order in which they appeared in the discrete-choice experiment questions

The mean preference coefficient estimates and 95% CIs for respondents with SZ at each attribute level are displayed in Fig. 2. Preference coefficient estimates were generally ordered as expected, with better outcomes (i.e., better efficacy, less risk of sedation, no adverse effects) being preferred to worse outcomes.

Respondents with SZ did not differentiate their preferences for antipsychotic medications with 0, 4, or 7 lb of weight gain over 6 months but indicated that they wanted to avoid medications that were associated with 11 lb of weight gain over 6 months. Improving treatment efficacy was more important than avoiding weight gain in almost all scenarios; avoiding weight gain of 11 versus 4 lb and 11 versus 0 lb was, however, more important than achieving the smallest incremental increase in efficacy. In addition, there was no differentiation between medications with a 10% risk of sedation versus those with no risk of sedation.

**Fig. 3:**
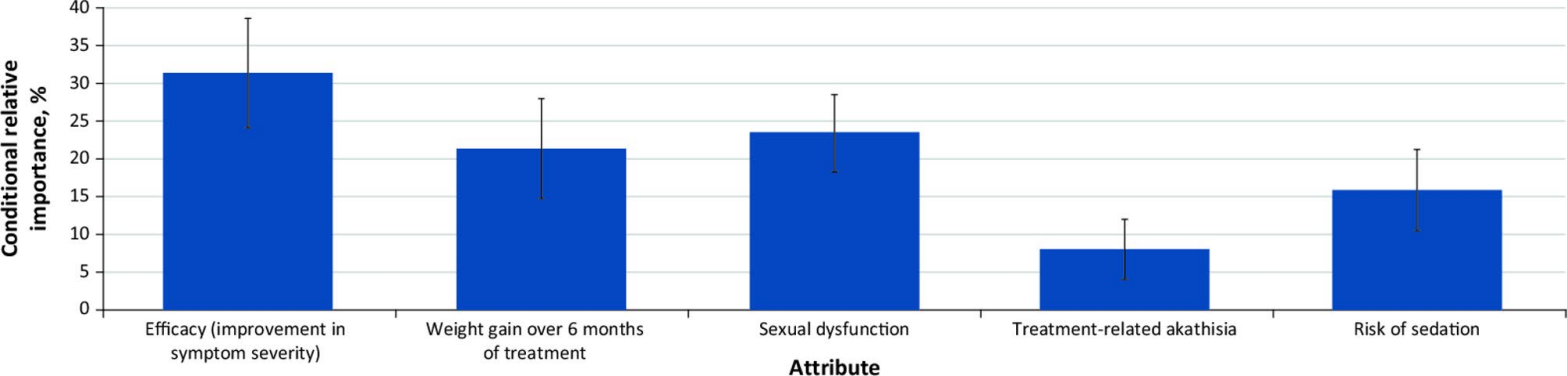
Relative importance of oral antipsychotic medication attributes for respondents with schizophrenia^**a**^ ^a^Conditional relative importance (CRI) is interpreted as the proportion of utility gained by improving each attribute from the least-preferred to the most-preferred level, relative to the maximum utility gained by improving all attributes. Each CRI was calculated by subtracting the preference coefficient estimate of the least-preferred level from that of the most-preferred level. Differences were summed across attributes and rescaled to 100. Each CRI is presented as a percentage of this total along with its 95% confidence interval. Attributes are presented in the order in which they appeared in the discrete-choice experiment questions

According to CRI estimates, treatment efficacy (CRI = 31.4%) was the most important attribute endorsed by respondents with SZ (Fig. 3). The second most important attribute was sexual dysfunction (CRI = 23.4%), followed by weight gain (CRI = 21.3%), risk of sedation (CRI = 15.9%), and akathisia (CRI = 8.0%).

With respect to MAWG, in order to achieve 1 incremental level of disease severity improvement, respondents with SZ were willing to accept a weight increase of 9.3 lb for “some” to “a lot” of improvement and 9.8 lb for “a little” to “some” improvement, as calculated using the formula described above. However, for 2 incremental steps of disease improvement (i.e., going from “a little” to “a lot” of improvement in efficacy), respondents were willing to accept a weight gain of > 11 lb over 6 months. On average, respondents were amenable to a MAR of > 25% for sedation for any level of symptom improvement.

#### Bipolar I disorder

**Fig. 4:**
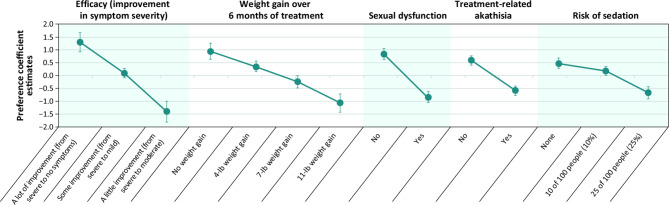
Preferences for attributes of oral antipsychotic medications for respondents with bipolar I disorder^**a**^ ^a^Preference coefficient estimates are presented along with their 95% confidence intervals, with higher estimates for a given level associated with a greater preference for that level. The vertical distance between any 2 levels of an attribute represents the change in utility; larger differences indicate that respondents viewed the change as having a relatively greater effect on overall utility. Attributes are presented in the order in which they appeared in the discrete-choice experiment questions

In respondents with BD-I, results were similar to those from respondents with SZ. Mean preference coefficient estimates for each attribute level are displayed in Fig. 4. Preference coefficient estimates for respondents with BD-I were generally ordered as expected, with better outcomes (i.e., better efficacy, no adverse effects, less risk of sedation) being preferred to worse outcomes.

For respondents with BD-I, avoiding weight gain of 11 versus 7 lb over 6 months was more important than avoiding weight gain of 7 versus 4 lb or of 4 versus 0 lb over 6 months. As with SZ, respondents with BD-I most wanted to avoid weight gain of 11 lb over 6 months. However, the largest improvement in symptom severity (from “a little” to “a lot”) was more important than avoiding weight gain.

**Fig. 5:**
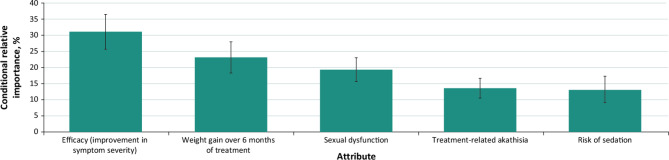
Relative importance of oral antipsychotic medication attributes for respondents with bipolar I disorder^**a**^ ^a^Conditional relative importance (CRI) is interpreted as the proportion of utility gained by improving each attribute from the least-preferred to the most-preferred level, relative to the maximum utility gained by improving all attributes. Each CRI was calculated by subtracting the preference coefficient estimate of the least-preferred level from that of the most-preferred level. Differences were summed across attributes and rescaled to 100. Each CRI is presented as a percentage of this total along with its 95% confidence interval. Attributes are presented in the order in which they appeared in the discrete-choice experiment questions

According to CRI estimates, treatment efficacy (CRI = 31.0%) was the most important attribute endorsed by respondents with BD-I (Fig. 5). The second most important attribute was weight gain (CRI = 23.1%), followed by sexual dysfunction (CRI = 19.2%), akathisia (CRI = 13.5%), and risk of sedation (CRI = 13.1%).

With respect to MAWG, in order to achieve 1 incremental level of disease severity improvement, respondents with BD-I were willing to accept a weight increase of 7.1 lb for “some” to “a lot” of improvement or 8.5 lb for “a little” to “some” improvement. As observed for respondents with SZ, in order to achieve 2 incremental levels of disease severity improvement (i.e., from “a little” to “a lot” of efficacy improvement), respondents were willing to accept a weight gain of > 11 lb over 6 months. On average, respondents were amenable to a MAR of > 25% for sedation for any level symptom improvement.

## Discussion

In this DCE eliciting preferences for attributes associated with oral antipsychotic medications, reducing symptoms associated with the respective disease state was considered by respondents with SZ or BD-I to be the most important attribute. Respondents also preferred oral antipsychotic medications with lower propensity for weight gain, a lack of treatment-related sexual dysfunction or akathisia, and a low risk of sedation. Avoiding weight gain and sexual dysfunction were the most important safety and tolerability considerations among both groups. However, respondents were still willing to accept some degree of weight gain for incremental improvements in antipsychotic efficacy, albeit with different tolerances for acceptable weight gain between respondents with SZ and those with BD-I based on the magnitude of improvement. For example, respondents with SZ did not differentiate between antipsychotic medications with no weight gain versus those with 4 or 7 lb of weight gain. Respondents with BD-I, however, showed a more linear pattern of weight gain avoidance; reducing the potential for weight gain from 11 to 7 lb was more important than reducing it from 7 to 4 lb, which in turn was more important than reducing it from 4 to 0 lb. These results suggest that individuals living with SZ or BD-I are willing to accept some degree of adverse effects, such as weight gain, if their antipsychotic medication provides better symptom control. Interestingly, respondents were willing to take an oral antipsychotic that causes sedation in 25 of 100 patients for *any* degree of disease state symptom improvement.

Previous studies using stated-preference methodology in patients with SZ or BD-I have reported that symptom improvement is among the most valued attributes of antipsychotic medications, while weight gain and adverse metabolic effects are among the most important adverse effects that patients desire to avoid [9–13]. Our results align with those of previous studies in this regard and provide additional insight into specific tradeoffs and the levels of tradeoffs that individuals are willing to accept [9, 11]. This has been observed in large effectiveness trials where differences in antipsychotic efficacy can drive continuing a medication despite the occurrence of weight gain [23].

These and other results from this analysis provide important information for clinicians and help emphasize the importance of clearly articulating to their patients the benefit and risk tradeoffs associated with specific antipsychotic medications in an effort to gauge patient preferences and adverse effect acceptability among individual patients. Because individual treatment responses vary, clinicians should consider the individual patient’s history of therapeutic response and adverse effects experienced when applying information derived from DCEs to clinical practice, regardless of clinical trial group data that may suggest differences between agents in terms of efficacy and tolerability [3], because individual patient preferences will differ.

Some limitations of this work should be noted. While survey respondents had a self-reported diagnosis of SZ or BD-I, the diagnosis was not confirmed by clinician assessment. In addition, the DCE tested a MAR of sedation of only 25%, so the relative importance of this adverse effect may be underestimated; higher sedation risk levels were not tested. A limitation inherent to stated-preference methodology is that respondents may overstate the value of attributes in response to a hypothetical scenario [24]. The focus of this DCE was on attributes of oral antipsychotics; therefore, indirect comparisons with DCEs focused on the attributes of long-acting injectable formulations should be approached with caution. Also, the results obtained in this study may not be generalizable to all patients with SZ or BD-I. Last, although this study provides valuable insights into patient preferences for antipsychotic medications, a patient’s clinical course and individual treatment experiences may significantly affect their response. Because of sample-size limitations, we were unable to adequately explore subgroup variations in the current analysis.

## Conclusions

Choosing between antipsychotic medications involves making tradeoffs between benefits and risks or burdens. Overall, respondents prioritized an antipsychotic medication that improved their symptoms of SZ or BD-I. Respondents were willing to accept modest weight gain (between 7 and 9 lb over 6 months) but not large weight changes (11 lb over 6 months) to achieve improvements in efficacy. The results of this study could be used to facilitate shared decision making, which may encourage clinicians to prescribe treatments that match their patients’ preferences.

## Electronic supplementary material

Below is the link to the electronic supplementary material.

Additional file 1: Identification of stated-preference studies

Additional file 2: Search strategy for the systematic literature review

Additional file 3: DCE survey instrument questions

Additional file 4: Analysis of preference data

## Data Availability

The data collected in this study are proprietary to Alkermes, Inc. Alkermes, Inc., is committed to public sharing of data in accordance with applicable regulations and laws.

## Abbreviations

BD-I: Bipolar I disorder
CI: Confidence interval
CRI: Conditional relative importance
DCE: Discrete-choice experiment
ISPOR: International Society for Pharmacoeconomics and Outcomes Research
MAR: Maximum acceptable risk
MAWG: Maximum acceptable weight gain
RPL: Random-parameter logit
SZ: Schizophrenia
