# Supplementary material for: Preferences for attributes of oral antipsychotic treatments: results from a discrete-choice experiment in respondents with schizophrenia or bipolar I disorder
Source: BMC Psychiatry. 2024 Sep 10;24:605. doi: 10.1186/s12888-024-06034-1 (PMC11389064; doi:10.1186/s12888-024-06034-1)
Supplement: Supplementary file 1 — Additional file 1: Identification of stated-preference studies [file 12888_2024_6034_MOESM1_ESM.pdf]

Additional File 1. Identification of stated-preference studies

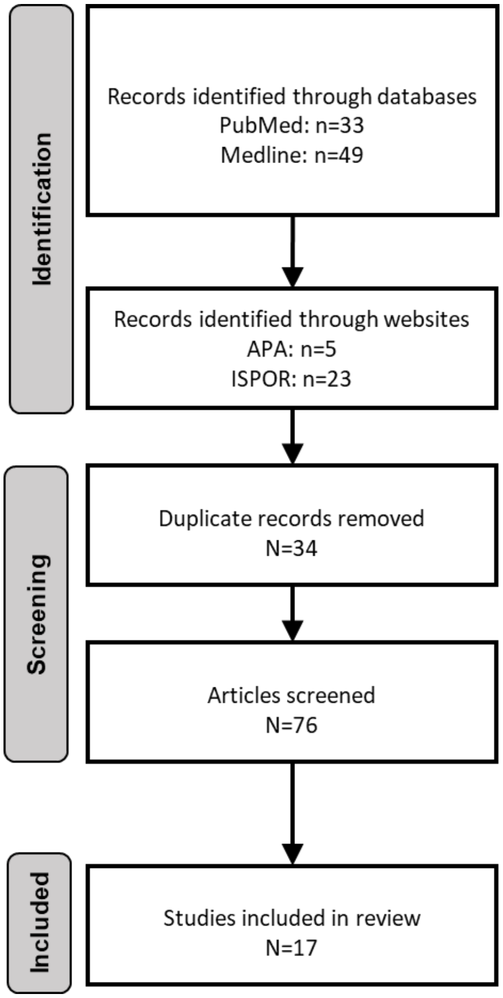

APA, American Psychiatric Association; ISPOR, International Society for Pharmacoeconomics and Outcomes Research.
