# Supplementary material for: Preferences for attributes of oral antipsychotic treatments: results from a discrete-choice experiment in respondents with schizophrenia or bipolar I disorder
Source: BMC Psychiatry. 2024 Sep 10;24:605. doi: 10.1186/s12888-024-06034-1 (PMC11389064; doi:10.1186/s12888-024-06034-1)
Supplement: Supplementary file 2 — Additional file 2: Search strategy for the systematic literature review [file 12888_2024_6034_MOESM2_ESM.pdf]

Additional File 2. Search strategy for the systematic literature review

A systematic literature search was conducted for attribute-based stated-preference studies published between January 1, 1990, and April 23, 2021, in the PubMed and Medline databases using the following keywords: discrete choice experiment*; discrete choice model*; discrete choice conjoint; stated preference*; conjoint analysis; conjoint choice experiment*; best worst scaling; threshold technique; conditional logit; mixed logit; and random parameter* logit. These keywords were combined with the following terms related to schizophrenia, schizoaffective disorder, and bipolar disorder: schizophreni*, schizoaffective; psychosis; psychotic; antipsychotic*; bipolar; manic; mania; hypomani*; mental N2 disord*.
