# Supplementary material for: Preferences for attributes of oral antipsychotic treatments: results from a discrete-choice experiment in respondents with schizophrenia or bipolar I disorder
Source: BMC Psychiatry. 2024 Sep 10;24:605. doi: 10.1186/s12888-024-06034-1 (PMC11389064; doi:10.1186/s12888-024-06034-1)
Supplement: Supplementary file 3 — Additional file 3: DCE survey instrument questions [file 12888_2024_6034_MOESM3_ESM.pdf]

Health Survey

Thank you for agreeing to take part in this health survey. We appreciate your time.

In this survey we are going to ask questions about your experience with [schizophrenia / bipolar disorder] and the treatments used for [schizophrenia / bipolar disorder].

Your Experience with Treatment for [Schizophrenia / bipolar disorder]

Let’s start with some questions about you and your health.

1. How old were you when a clinician or healthcare provider first diagnosed you with [schizophrenia / bipolar disorder] (Please use your best guess if you are not sure of the exact age)?

____

2. [If Condition = Schizophrenia] **Which medicines are you currently prescribed or have been prescribed for you in the past by a healthcare professional for schizophrenia? (Please *select all that apply.*)**

Type of Medicine	Currently Taking	Have Taken in the Past	Never Taken
**Oral first generation or typical antipsychotics** such as:<br>• chlorpromazine (Thorazine),<br>• fluphenazine (Prolixin),<br>• perphenazine (Trilafon),<br>• thioridazine (Mellaril),<br>• trifluoperazine (Stelazine),<br>• haloperidol (Haldol),<br>• thiothixene (Navane),<br>• loxapine (Loxitane)	☐	☐	☐
**Oral second generation or atypical antipsychotics** such as:<br>• aripiprazole (Abilify),<br>• asenapine (Saphris),<br>• cariprazine (Vraylar),<br>• clozapine (Clozaril),<br>• iloperidone (Fanapt),<br>• lurasidone (Latuda),<br>• olanzapine (Zyprexa),<br>• olanzapine/samidorphan (LYBALVI),<br>• quetiapine (Seroquel),<br>• risperidone (Risperdal),<br>• ziprasidone (Geodon),<br>• lumateperone (Caplyta),<br>• brexpiprazole (Rexulti)	☐	☐	☐
**Long-acting injectable antipsychotics** such as:<br>• aripiprazole (Abilify Maintena),<br>• aripiprazole lauroxil (Aristada),<br>• olanzapine pamoate (Zyprexa	☐	☐	☐

Type of Medicine	Currently Taking	Have Taken in the Past	Never Taken
Relprevv),<br>• paliperidone (Invega Sustenna, Invega Trinza, Invega Hafyera),<br>• risperidone (Risperdal Consta)			
**Antidepressants** such as:<br>• sertraline (Zoloft),<br>• vortioxetine (Trintellix),<br>• fluoxetine (Prozac),<br>• paroxetine (Paxil),<br>• duloxetine (Cymbalta),<br>• venlafaxine (Effexor)	☐	☐	☐
**Antianxiety medications** such as:<br>• diazepam (Valium),<br>• lorazepam (Ativan),<br>• clonazepam (Klonopin),<br>• oxazepam (Serax),<br>• chlordiazepoxide (Librium),<br>• alprazolam (Xanax)	☐	☐	☐
**Other**	☐	☐	☐

3. **[If Condition=Bipolar disorder] Which medications are you currently prescribed or have been prescribed for you in the past by a healthcare professional for bipolar disorder? (Please *select all that apply.*)**

Type of Medication	Currently Taking	Have Taken in the Past	Never Taken
**Oral first generation or typical antipsychotics** such as<br>• chlorpromazine (Thorazine),<br>• fluphenazine (Prolixin),<br>• thioridazine (Mellaril),<br>• haloperidol (Haldol)	☐	☐	☐
**Oral second generation or atypical antipsychotics** such as<br>• aripiprazole (Abilify),<br>• clozapine (Clozaril),<br>• olanzapine (Zyprexa), olanzapine/samidorphan (Lybalvi),<br>• quetiapine (Seroquel),<br>• risperidone (Risperdal),<br>• ziprasidone (Geodon),<br>• zotepine (Zoleptil)	☐	☐	☐
**Long-acting injectable antipsychotics** such as<br>• aripiprazole (Abilify Maintena),<br>• risperidone (Risperdal Consta)	☐	☐	☐
**Mood stabilizers** such as<br>• lithium (Lithobid),<br>• valproic acid (Depakene),<br>• divalproex sodium (Depakote),<br>• carbamazepine (Tegretol, Equetro),<br>• lamotrigine (Lamictal)	☐	☐	☐
**Antidepressants** such as:<br>• sertraline (Zoloft),<br>• vortioxetine (Trintellix),<br>• fluoxetine (Prozac),	☐	☐	☐

Type of Medication	Currently Taking	Have Taken in the Past	Never Taken
• paroxetine (Paxil),<br>• duloxetine(Cymbalta),<br>• venlafaxine (Effexor)			
**Antianxiety medications** such as<br>• diazepam (Valium),<br>• lorazepam (Ativan),<br>• clonazepam (Klonopin),<br>• oxazepam (Serax),<br>• chlordiazepoxide (Librium),<br>• alprazolam (Xanax)	☐	☐	☐
**Other**	☐	☐	☐

4. Have you ever received any of the following treatments?
(Please select all that apply.)

- ☐ Psychotherapy/counseling (including individual or group therapy/counseling, cognitive behavioral therapy [CBT], and/or interpersonal and social rhythm therapy [IPSRT])
- ☐ Electroconvulsive therapy (ECT)
- ☐ Transcranial magnetic stimulation (TMS)
- ☐ Ketamine treatment or esketamine (Spravato) nasal spray
- ☐ Other therapies
- ☐ None of the above [Exclusive]

[If Condition = Schizophrenia]

Schizophrenia Symptoms and Symptoms Severity

In the next few pages we are going to discuss schizophrenia symptoms and symptoms severity.

People with schizophrenia may describe their symptoms as follows:

1. **Severe symptoms:**

You hear voices and have very bothersome thoughts that you feel like you need to act on. People frequently find it difficult to follow what you are saying, and you find it difficult to express yourself. You regularly have trouble thinking and understanding things that you would normally be able to understand. Often, you feel hopeless, guilty, and worried. Other times you may feel unusually excited and restless. You feel these "ups" and "downs" daily.

2. **Moderate symptoms:**

You frequently have bothersome thoughts and hear voices. Sometimes people find it difficult to follow what you are saying, and you sometimes find it difficult to express yourself. You sometimes feel foggy and confused. You still feel "ups" and "downs" but not every day.

3. **Mild symptoms:**

You might sometimes hear voices or have thoughts that others may find "strange", but these are not very bothersome and you can easily ignore them. Most people can follow what you are saying, and you are generally able to express yourself. You are usually able to understand and think about things without feeling too confused. Sometimes you feel unusually high, excited, and restless but not often.

4. **No symptoms:**

Your symptoms are very much improved, and now you have no schizophrenia symptoms and you are able to go about daily life without any impact on behavior.

You will experience no symptoms as long as you take the medicine. After 6 months, you are said to be in "recovery."

5. How would you describe your schizophrenia symptoms at their best in the past week?

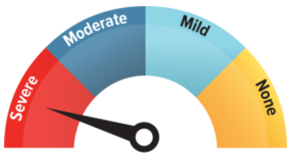	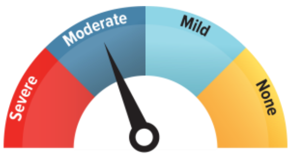	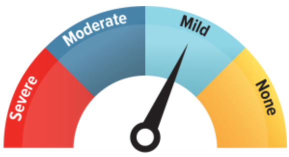	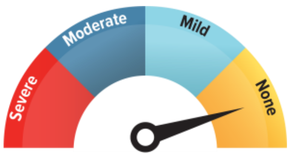
**Severe symptoms**<br>(Heard voices and had **very bothersome** thoughts and the urge to act on them)	**Moderate symptoms**<br>(**Frequently** had **bothersome** thoughts and heard voices that are difficult to ignore)	**Mild symptoms**<br>(**Sometimes** heard voices or had **strange thoughts**, but these were not too bothersome)	**No symptoms**<br>(**No symptoms** and was able to go about daily life **with NO impact** on behavior)
☐	☐	☐	☐

6. [If Q5 is not Severe, show question] [Show all response options equal to or greater in severity than response to Q5] How would you describe your schizophrenia symptoms at their worst in the past week?

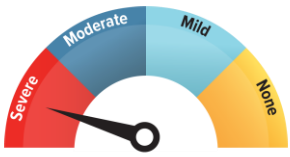	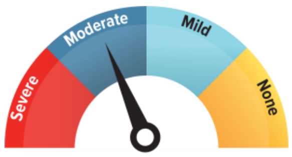	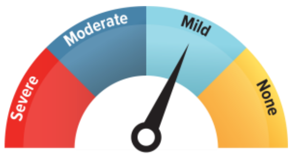	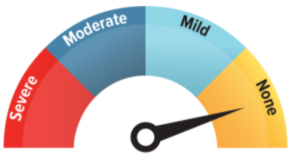
**Severe symptoms**<br>(Heard voices and had **very bothersome** thoughts and the urge to act on them)	**Moderate symptoms**<br>(**Frequently** had bothersome thoughts and heard voices that were difficult to ignore)	**Mild symptoms**<br>(**Sometimes** heard voices or had strange thoughts, but these were not too bothersome)	**No symptoms**<br>(**No symptoms** and was able to go about daily life **with NO impact** on behavior)
☐	☐	☐	☐

[If Condition = Bipolar disorder]

Bipolar Disorder Symptoms and Symptoms Severity

In the next few pages we are going to discuss Bipolar disorder symptoms and symptoms severity.

People with bipolar disorder may describe their symptoms as follows:

1. **Severe symptoms:**

You have episodes of feeling out of touch with the world around you as if nothing feels real. You feel you do not need to sleep as much as before. Your behavior becomes impulsive and you start to take risks such as spending money and or driving too fast/recklessly. You have lots of ideas, but these are unrealistic.

Other people noticed the change in your behavior, but you didn’t.

2. **Moderate symptoms:**

You feel excited and full of energy and have ideas about new projects and adventures, but these ideas are more realistic and achievable. However, you then have to slow down, you may feel frustrated that some people can’t keep up with your conversation or plans. In response, you can find yourself being sarcastic and short with others, even with your friends and family.

Both you and others recognized the change in your behavior.

3. **Mild symptoms:**

You have “ups,” where you feel talkative and over-excited.

You and a few close friends and family can notice a change in your behavior but others, generally, do not.

4. **No symptoms:**

You have no symptoms; you are able to go about your day-to-day life as there is little-to-no change in your behavior.

7. **How would you describe your bipolar disorder symptoms at their <u>best</u> in <u>the past week</u>?**

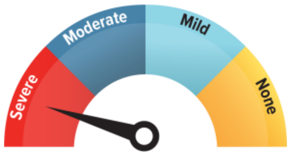	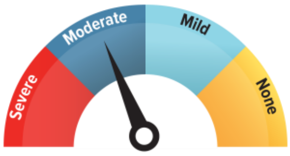	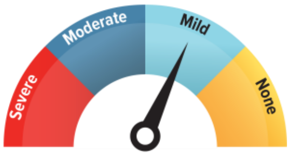	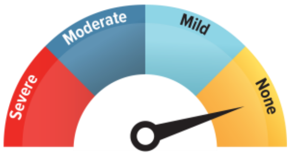
**Severe symptoms**<br>(In your "ups," you had lots of energy and ideas, but these were unrealistic. Other people noticed the change in your behavior, but you didn't.)	**Moderate symptoms**<br>(In your "ups," you had lots of energy and ideas and were excited about new ventures. Both you and others recognized the change in your behavior.)	**Mild symptoms**<br>(In your "ups," you felt talkative and over-excited. You and a few close friends and family noticed the change in your behavior.)	**No symptoms**<br>(You had no symptoms; you were able to go about your day-to-day life as there was little-to-no change in your behavior.)
☐	☐	☐	☐

8. **[If Q7 is not Severe, show question] [Show all response options equal to or greater in severity than response to Q7]** **How would you describe your bipolar disorder symptoms at their <u>worst</u> in <u>the past week</u>?**

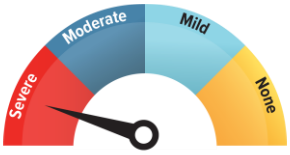	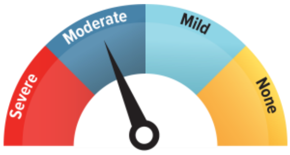	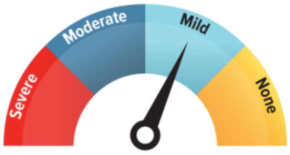	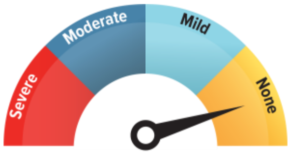
**Severe symptoms**<br>(In your "ups," you had lots of energy and ideas, but these ideas were unrealistic. Other people noticed the change in your	**Moderate symptoms**<br>(In your "ups," you had lots of energy and ideas and were excited about new ideas. Both you and others recognized the change in your behavior.)	**Mild symptoms**<br>(In your "ups," you felt talkative and over-excited. You and a few close friends and family noticed the change in your behavior.)	**No symptoms**<br>(You had no symptoms; you were able to go about your day-to-day life as there was little-to-no change in your behavior.)

behavior, but you didn’t.)			
☐	☐	☐	☐

Characteristics of Medicines for [Schizophrenia / bipolar disorder]

[If Condition = Schizophrenia]

Doctors prescribe treatments to stop or decrease the symptoms of schizophrenia. Treatment often involves taking medicines called antipsychotics that can reduce delusions (for example, feeling of paranoia) and hallucinations (for example, hearing voices).

[If Condition = Bipolar disorder]

Doctors prescribe treatments to stop or prevent symptoms of bipolar disorder. Treatment often involves taking medicines called antipsychotics that can help with the “ups” and the “downs”, and to prevent episodes of “ups” and “downs” from recurring.

All respondents

In this survey, we are interested in what you think about different antipsychotic medicines that doctors may prescribe to help manage your [schizophrenia / bipolar disorder] symptoms.

Over the next few pages, we will ask you to think about different hypothetical antipsychotic medicines with different characteristics. These characteristics include:

- Improvement in symptoms
- Weight gain in the 6 months after starting the medicine
- Problems with Sexual Functioning
- Feeling restless and “can’t sit still”
- Risk of drowsiness or feeling tired

All of the hypothetical medicines are taken by mouth once a day for as long as they help you keeping your severe symptoms under control.

Please read these descriptions carefully. This information will help you answer questions later in the survey.

[If Condition = Schizophrenia]

Medicine Characteristic: Improvement in Symptoms

In this survey, we would like you to imagine that your current medicine is no longer working and that your schizophrenia symptoms have become severe and have started to interfere with your everyday life and your ability to do your daily activities.

For example, it can be hard to concentrate on regular activities like taking care of yourself (washing, cooking) or going out (to shop or see friends and family), and almost impossible for you to fully concentrate on your work or studies (miss deadlines, unable to complete assigned tasks).

Please remember, with these severe symptoms:

- You hear voices and have very bothersome thoughts that you feel like you need to act on.
- People frequently find it difficult to follow what you are saying, and you find it difficult to express yourself.
- You regularly also have trouble thinking and understanding things that you would normally be able to understand.
- Other times you might feel unusually excited and restless. You feel these “ups” and “downs” daily.

Suppose you feel like this, and your doctor recommends you try a new medicine. Some of the medicines we will ask you to think about are more effective at controlling your symptoms than others.

In this survey, we will ask you to think about hypothetical medicines that may be more or less effective in improving your severe symptoms. The improvements in symptoms we want you to consider are summarized in the table below.

**Improvement in Symptoms**	
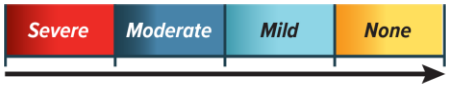<br>**A lot of improvement**<br>From severe to no symptoms	**Severe symptoms improve a lot:**<br>• You have no symptoms, and your schizophrenia is not impacting your behavior or your day-to-day life.<br>• You will experience no symptoms as long as you take the medicine; after 6 months, you are said to be in "recovery."
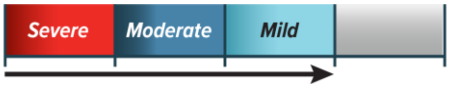<br>**Some improvement**<br>From severe to mild	**Severe symptoms improve considerably:**<br>• Sometimes, you hear voices or have strange thoughts, but these are not very bothersome and you can easily ignore them.<br>• Most people can follow what you are saying, and you are generally able to express yourself.<br>• You don't feel foggy or confused and you are usually able to understand and think about things.<br>Sometimes you feel unusually excited and restless but not often.
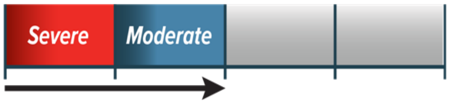<br>**A little improvement**<br>From severe to moderate	**Severe symptoms improve a little:**<br>• Frequently, you have bothersome thoughts and hear voices.<br>• Sometimes people find it difficult to follow what you are saying, and you sometimes find it difficult to express yourself.<br>• You sometimes feel foggy and confused.<br>• You still feel "ups" and "downs" but not every day.

[If Condition = Bipolar disorder]

Medicine Characteristic: Improvement in Symptoms

In this survey, we would like you to imagine that your current medicine is no longer working and that your manic symptoms have become severe and have started to interfere with your everyday life and your ability to do your daily activities.

For example: it can be hard to concentrate on regular activities like taking care of yourself (washing, cooking) or going out (to shop or see friends and family), and almost impossible for you to fully concentrate on your work or studies.

Please remember, with these severe symptoms:

- You have episodes of feeling out of touch with the world around you as if nothing feels real. You feel you do not need to sleep as much as before. Your behavior becomes impulsive, and you start to take risks such as spending money and or driving too fast/recklessly.
- Your friends and family find it difficult to keep up with you or follow what you are doing or saying, but you don't want to pause or be interrupted which can cause arguments. Although others have noticed or commented on your behavior, you don't believe you are acting differently.
- You have lots of ideas, but these are unrealistic.
- Other people notice the change in your behavior, but you don't.

Suppose you feel like this, and your doctor recommends you try a new medicine. Some of the medicines we will ask you to think about are more effective at controlling your symptoms than others.

In this survey, we will ask you to think about hypothetical medicines that may be more or less effective in improving your severe symptoms. The improvements in symptoms we want you to consider are summarized in the table below.

**Improvement in Symptoms**	
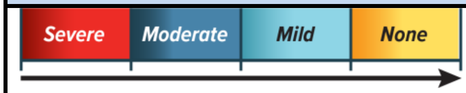<br>**A lot of improvement**<br>From <u>severe</u> to <u>no symptoms</u>	**Severe symptoms improve a lot:**<br>• You experience no symptoms impacting your behavior or your day-to-day life.<br>• This will last as long as you take the medicine; after 8 weeks, you will said to be in "recovery.
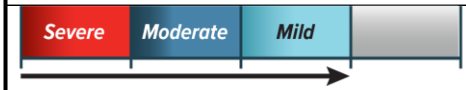<br>**Some improvement**<br>From <u>severe</u> to <u>mild</u>	**Severe symptoms improve considerably:**<br>• Your manic periods are milder, although you are noticeably active and over-excited.<br>• You still have "ups" and "downs," but your "ups" feel less extreme.<br>• When others have commented on your behavior, you realize that you are very active and perhaps over-excited.
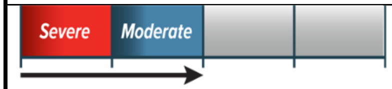<br>**A little improvement**<br>From <u>severe</u> to <u>moderate</u>	**Severe symptoms improve a little:**<br>• Your manic periods are a little less extreme although you still feel full of energy. Your ideas are more realistic and achievable<br>• Although you have slowed down, you may feel frustrated that some people can't keep up with your conversation or plans. In response, you can find yourself being sarcastic and short with others, even with your friends and family.<br>• Both you and others recognize the change in your behavior.

Medicine Characteristic: Weight Gain in the 6 Months After

Starting the Medicine

Weight gain has been observed in people taking medicines similar to the hypothetical medicines we are discussing in this survey. Some of the hypothetical medicines we are discussing are linked to a higher weight gain than others.

9. Have you <u>ever</u> experienced weight gain because of a medicine you have ever taken for [schizophrenia / bipolar disorder] ?

- ☐ Yes
- ☐ No
- ☐ Don’t know or not sure

If Condition = Schizophrenia

The weight gain may mean that you feel more uncomfortable, as your clothes may not fit as well as they did before you started the medicine. You may also start to worry about your appearance which affects your self-esteem.

In this survey, we will ask you to think about weight gain due to the hypothetical medicine rather than weight gain due to over-eating or eating foods that may lead to weight gain.

If Condition = Bipolar disorder

The weight gain may mean that you feel more uncomfortable, as your clothes may not fit as well as they did before you started the medicine. You may also start to worry about your appearance which affects your self-esteem.

In this survey, we will ask to think about weight gain due to the hypothetical medicine rather than weight gain due to over-eating, eating foods that may lead to weight gain or to your bipolar disorder symptoms. However, we will ask some questions about your experience with weight gain due to actual medicines you have taken.

10. **[If Q9 = yes] What is the most weight you have gained in a 6 month period due to a medicine for [schizophrenia / bipolar disorder]?**

_______________ lbs.

In this survey, we will ask you to think about 4 possible amounts of weight gain in the 6 months after starting the hypothetical medicine.

**Weight Gain in the 6 Months After Starting the Medicine**	
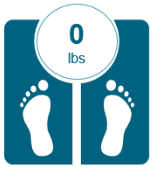 **No weight gain**	You do not gain any weight because of the medicine.
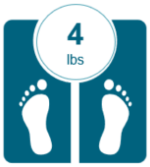 **4 lb weight gain**	The medicine causes your weight to increase by 4 lbs in the 6 months after starting it.
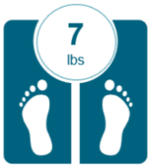 **7 lb weight gain**	The medicine causes your weight to increase by 7 lbs in the 6 months after starting it.
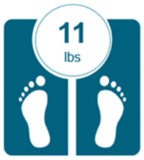 **11 lb weight gain**	The medicine causes your weight to increase by 11 lbs in the 6 months after starting it.

[Comprehension Q1]

11. Based on the table below, which medicine offers the biggest improvement in your [schizophrenia/bipolar disorder] symptoms?

- ☐ Medicine A
- ☐ Medicine B
- ☐ Don't know or not sure

**Characteristic**	**Medicine A**	**Medicine B**
**Improvement in symptoms after starting the medicine**	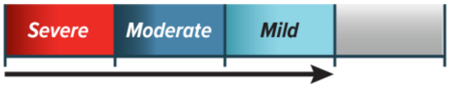<br>**Some improvement**<br>From severe to mild	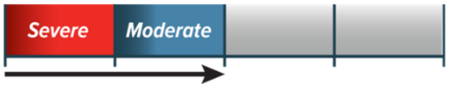<br>**A little improvement**<br>From severe to moderate
**Weight gain in the 6 months after starting the medicine**	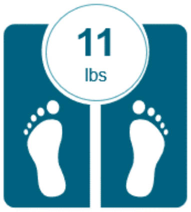<br>11 lb weight gain	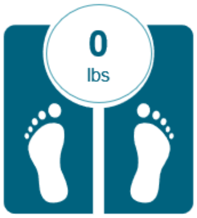<br>No weight gain

[If Q11 = Medicine A]

Characteristic	Medicine A	Medicine B
Improvement in symptoms after starting the medicine	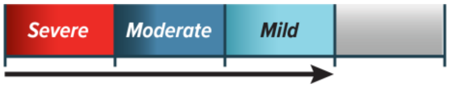<br>**Some improvement**<br>From severe to mild	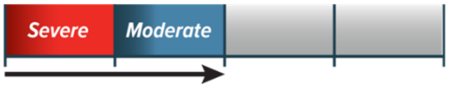<br>**A little improvement**<br>From severe to moderate
Weight gain in the 6 months after starting the medicine	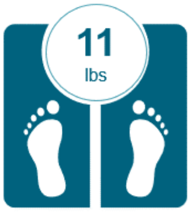<br>11 lb weight gain	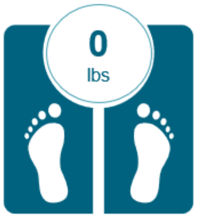<br>No weight gain

You are correct. With Medicine A, symptoms are much improved. With Medicine B, symptoms are minimally improved.

[If Q11 ≠ Medicine A]

**Characteristic**	**Medicine A**	**Medicine B**
Improvement in symptoms after starting the medicine	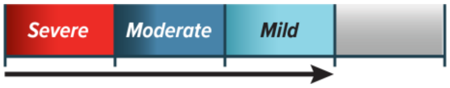 **Some improvement**<br>From <u>severe</u> to <u>mild</u>	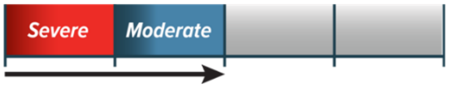 **A little improvement**<br>From <u>severe</u> to <u>moderate</u>
Weight gain in the 6 months after starting the medicine	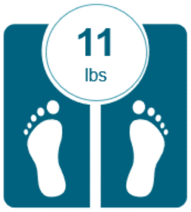 11 lb weight gain	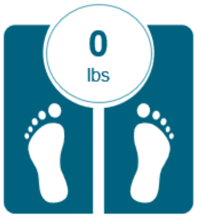 No weight gain

Remember, in this survey, we want you to imagine that your current medicine is no longer working and that your manic symptoms are severe and have started to interfere with your everyday life and your ability to do your daily activities. The two hypothetical medicines give you two different improvements.

- With Medicine A, symptoms are much improved, from severe to mild.
- With Medicine B, symptoms are minimally improved, from severe to moderate.

Therefore, the correct answer is medicine A

Medicine Characteristic: Problems with Sexual Functioning Because of the Medicine

Some of the hypothetical medicines can cause changes in hormones that may affect how you feel and act about or during sex.

Some people will feel no change in their feelings about or performance with sexual activity. For others, they may feel their sex drive reduce as they become disinterested in sex. In some cases, the medicine may prevent your ability to participate in sexual activities, as you may not become physically aroused.

If you experience this side effect, it will last as long as you take the medicine.

Later in this survey, we will ask you to think about hypothetical [schizophrenia/bipolar disorder] medicines that can affect your sexual functioning and medicines that do not affect your sexual functioning.

12. Have you ever experienced changes in your sex drive or sexual functioning because of a medicine you have taken for your [schizophrenia/bipolar disorder]? ***(Please select all that apply.)***

- ☐ Yes, I experienced lack of interest in sexual activity
- ☐ Yes, I experienced inability to participate in sexual activity
- ☐ No, I have never experienced either [Exclusive]
- ☐ Don’t know or not sure [Exclusive]

Medicine Characteristic: Feeling Restless Because of the Medicine

Some medicines can cause you to feel restless and make you feel like you "can't sit still". If you experience restlessness because of the medicine, you may find it difficult to stay still. You may find yourself fidgeting or twitching. The urge to move may be something that you cannot easily control.

You may also feel tense or a little annoyed. These feelings are because of the medicine, rather than your [schizophrenia/bipolar disorder], and will last as long as you take the medicine.

In this survey, we will ask you to think about medicines that cause restlessness and medicines that do not cause restlessness.

13. Have you ever experienced restlessness, as described above, because of a medicine you have taken for your [schizophrenia/ bipolar disorder]?

- ☐ Yes
- ☐ No
- ☐ Don't know or not sure

Medicine Characteristic: Risk of Feeling Drowsy or Tired Because of the Medicine

Some medicines can cause you to feel excessively sleepy and drowsy. Feeling drowsy may mean that you feel like you could fall asleep during the day as well as at night. You may also find it difficult to get up in the morning. People who experience drowsiness or excessive sleepiness often say that they feel “knocked out.”

If you feel drowsy or sleepy because of the medicine, you will find it difficult to do your usual day-to-day activities or do things that require you to be alert. For example, you will need to stop driving.

This drowsiness is caused by the medicine and will last as long as you take the medicine.

14. Have you ever experienced drowsiness because of a medicine you have taken for your [schizophrenia / bipolar disorder]?

- ☐ Yes
- ☐ No
- ☐ Don’t know or not sure

[Risk tutorial]

Thinking About the Risk of Feeling Drowsy or Tired Because of The Medicine

The following picture might help you think about the risk of feeling drowsy or tired because of the medicine.

There are 100 human-shape figures in the picture below. Each of these human-shape figure show 1 person who takes the medicine.

The figures in gray show the number of people who will not feel drowsy or tired because of the medicine.

The figures in blue show the number of people who will feeling drowsy or tired because of the medicine.

The more figures shown in blue, the higher the risk.

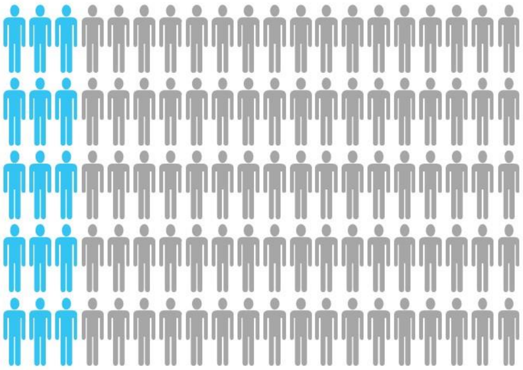

In this example:

15 of the figures are shown in blue. This means that 15 of 100 people (15%) will feel drowsy or tired because of the medicine.

85 of the figures are shown in gray. This means that 85 people out of 100 (85%) will not feel drowsy or tired because of the medicine.

[Risk practice question]

Please look at the picture below:

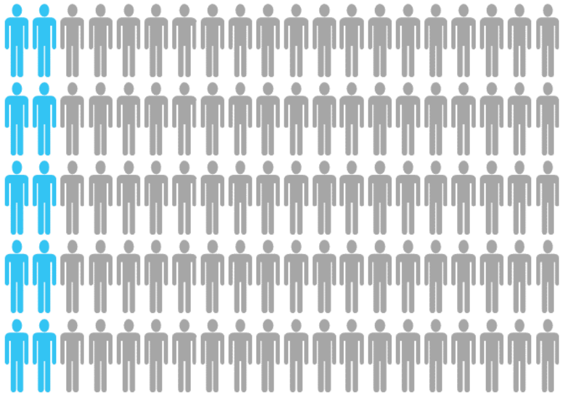

15. There are 100 human-shape figures in the picture and each one represents 1 person who takes the medicine and there are 10 figures in blue. How many people will feel drowsy or tired because of this medicine?

- ☐ 10 out of 100 people (10%)
- ☐ 20 out of 100 people (20%)
- ☐ 25 out of 100 people (25%)
- ☐ 90 out of 100 people (90%)

[If Q15 = 10 out of 100 (10%)]

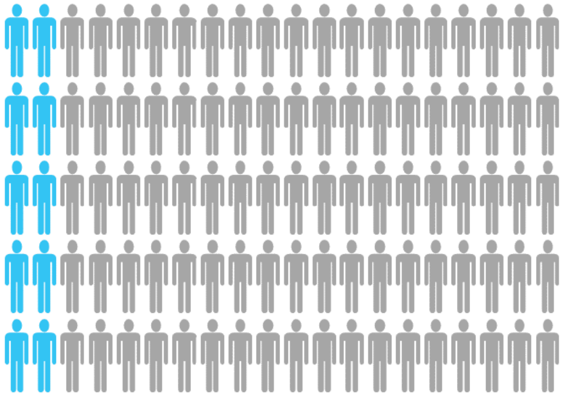

You are correct. There are 100 human-shape figures in the picture, and 10 of these figures are blue. The 10 figures in blue indicate that 10 people taking the medicine will feel drowsy or tired. Therefore, 10 of 100 people (10%) is the correct answer.

[If Q15 ≠ 10 out of 100 (10%)]

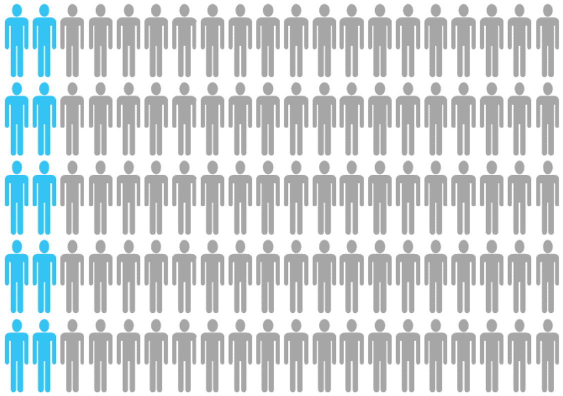

Based on your response, we want to remind you that each human-shape figure in the picture represents someone who is taking this medicine.

- There are 100 figures in the picture.
- 10 of these figures appear in blue. These 10 figures in blue indicate that 10 people taking the medicine will feel drowsy or tired.
- Therefore, 10 out of 100 people (10%) is the correct answer.

In this survey, we will ask you to think about 3 possibilities for the risk of feeling drowsy while taking the medicine.

**Risk of Feeling Drowsy or Tired Because of the Medicine**	
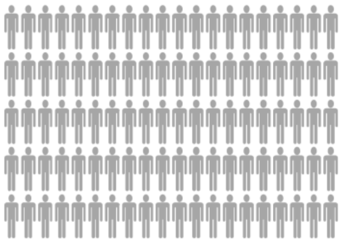 **None**	**None** of the people who take this medicine will feel drowsy or tired because of the medicine.
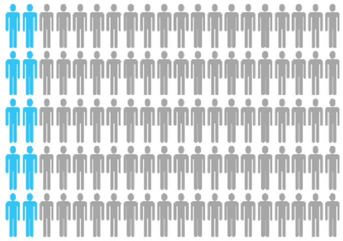 **10 out of 100 people (10%)**	**10 out of 100** people (10%) who take this medicine will feel drowsy or tired because of the medicine.
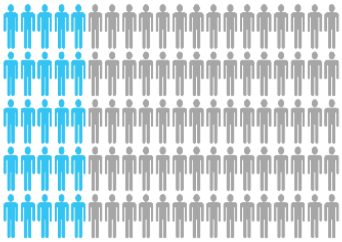 **25 out of 100 people (25%)**	**25 out of 100** people (25%) who take this medicine will feel drowsy or tired because of the medicine.

[Comprehension Q2]

16. Please look at the table below, based on the information included, which medicine has the highest risk of feeling drowsy or tired?

- ☐ Medicine A
- ☐ Medicine B
- ☐ Don't know or not sure

Medicine Characteristic	Medicine A	Medicine B
Improvement in symptoms after starting the medicine	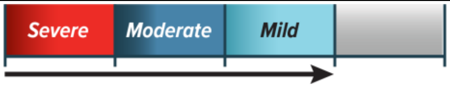 **Some improvement**<br>From <u>severe</u> to <u>mild</u>	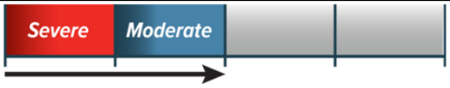 **A little improvement**<br>From <u>severe</u> to <u>moderate</u>
Weight gain in the 6 months after starting the medicine	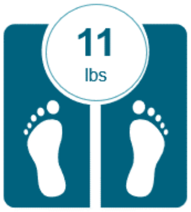 11 lb weight gain	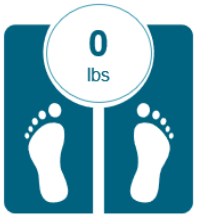 No weight gain
Problems with sexual functioning because of the medicine	Yes	No
Feeling restless because of the medicine	No	No
Risk of feeling drowsy or tired because of the medicine	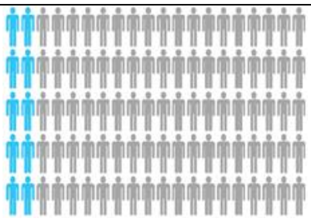 10 out of 100 people (10%)	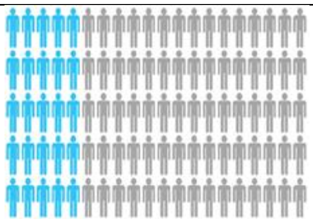 25 out of 100 people (25%)

[If Q16 = Medicine B]

**Medicine Characteristic**	**Medicine A**	**Medicine B**
Improvement in symptoms after starting the medicine	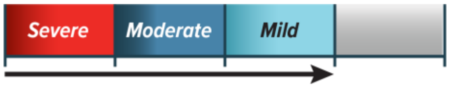 **Some improvement** From severe to mild	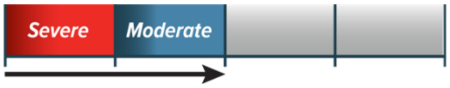 **A little improvement** From severe to moderate
Weight gain in the 6 months after starting the medicine	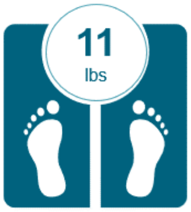 11 lb weight gain	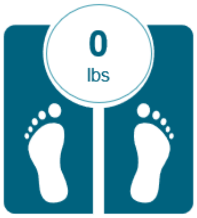 No weight gain
Problems with sexual functioning because of the medicine	**Yes**	**No**
Feeling restless because of the medicine	**No**	**No**
Risk of feeling drowsy or tired because of the medicine	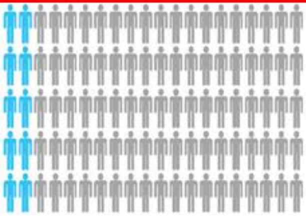 10 out of 100 people (10%)	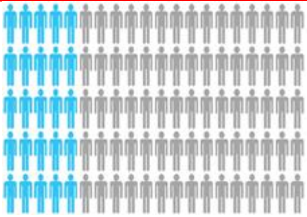 25 out of 100 people (25%)

You are correct.

With Medicine B, 25 out of 100 people (25%) taking the medicine will feel drowsy or tired because of this medicine, while with Medicine A 10 out of 100 people (10%) will feel drowsy or tired because of this medicine.

[If Q16 ≠ Medicine B]

Medicine Characteristic	Medicine A	Medicine B
Improvement in symptoms after starting the medicine	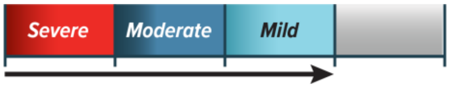<br>**Some improvement**<br>From severe to mild	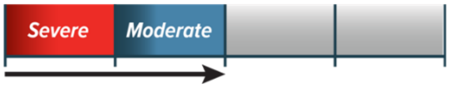<br>**A little improvement**<br>From severe to moderate
Weight gain in the 6 months after starting the medicine	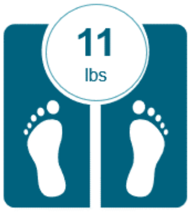<br>11 lb weight gain	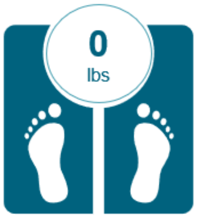<br>No weight gain
Problems with sexual functioning because of the medicine	**Yes**	**No**
Feeling restless because of the medicine	**No**	**No**
Risk of feeling drowsy or tired because of the medicine	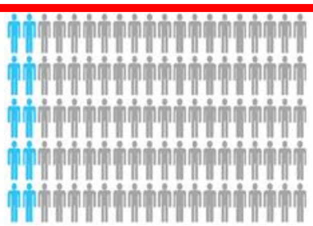<br>10 out of 100 people (10%)	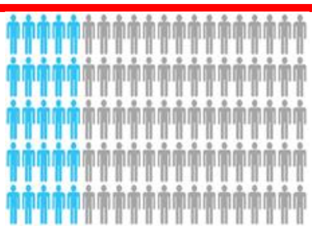<br>25 out of 100 people (25%)

Remember, with Medicine B 25 out of 100 people (25%) taking the medicine will feel drowsy or tired because of this medicine, while with Medicine A 10 out of 100 people (10%) will feel drowsy or tired because of this medicine.

Medicine B is correct.

Your Opinions About Medicines

Suppose that your current medicine is no longer working and that your symptoms have become severe and have started to interfere with your everyday life and your ability to do your daily activities.

[Respondent can see in popup the appropriate description of severe symptoms]

Your doctor recommends that you take one of the two alternative antipsychotic medicines (Medicine A and Medicine B). If you take other medicines, such as antidepressants, you would keep taking those medicines the same way you do now.

In the next 12 questions, we will show you different pairs of possible medicines. For each pair of medicines, please tell us which medicine you would prefer (Medicine A or Medicine B).

If you would like to see the description for any feature, feel free to move the mouse over that medicine feature to see the description again. [NOTE: this depends on how it is programmed online… please check that the sentence is accurate]

Please remember that:

- Even if you don’t like either option very much, please pick the 1 that you would choose if you had no other alternatives.
- The 2 options will be different in each question.
- Assume that both medicines have the same out-of-pocket cost.
- Assume that both medicines are taken by mouth (a pill) once a day for as long as they help you keeping your severe symptoms under control.

Please select one option considering your preference for all features described. There are no right or wrong answers.

[NOTE: Insert DCE here.

The following page includes an example DCE task. It is a placeholder and should not actually be shown to respondents.

Each respondent will be presented with 12 experimentally designed DCE tasks in random order.

[Example] Please look at the table below. If these were your only medicine options, which one would you choose? X/12

Characteristic	Medicine A	Medicine B
Improvement in symptoms after starting the medicine	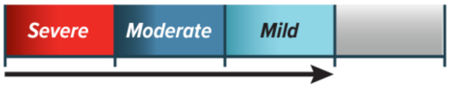<br>**Some improvement**<br>From <u>severe</u> to <u>mild</u>	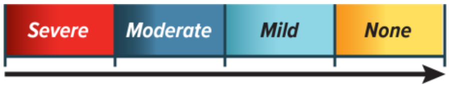<br>**A lot of improvement**<br>From <u>severe</u> to <u>no symptoms</u>
Weight gain in the 6 months after starting the medicine	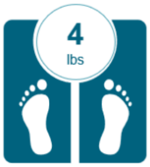<br>4 lb weight gain	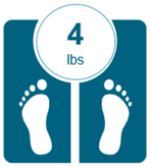<br>4 lb weight gain
Problems with sexual functioning because of the medicine	**Yes**	**No**
Feeling restless because of the medicine	**No**	**No**
Risk of feeling drowsy or tired because of the medicine	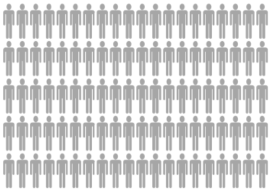<br>None	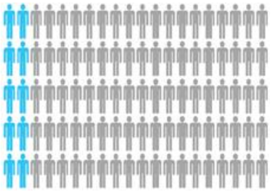<br>10 out of 100 people (10%)
**If you could only choose between these 2 medicines, which option would you choose?**	☐	☐

Questions About Your Experience with Side Effects

We would like to know about both the positive and negative experiences associated with medicine(s) you have ever used for [schizophrenia/bipolar disorder]. When responding, please think about all of the medicines that you have been on rather than any specific medicine.

17. When you think about all the medicine(s) you have ever taken for [schizophrenia / bipolar disorder], how bothersome have you found the following side effects?

	N/A Never experienced this side effect	Not bothersome	Somewhat bothersome	Very bothersome	Extremely bothersome
Digestive problems, including nausea and diarrhea	☐	☐	☐	☐	☐
Dizziness / fainting	☐	☐	☐	☐	☐
Dry mouth	☐	☐	☐	☐	☐
Feeling a lack of emotion	☐	☐	☐	☐	☐
Feeling drowsy or tired	☐	☐	☐	☐	☐
Feeling like a “zombie”	☐	☐	☐	☐	☐

	N/A Never experienced this side effect	Not bothersome	Somewhat bothersome	Very bothersome	Extremely bothersome
Involuntary muscle spasms, movements, twitching, or muscle stiffness	☐	☐	☐	☐	☐
Restlessness	☐	☐	☐	☐	☐
Sexual dysfunction	☐	☐	☐	☐	☐
Trouble concentrating	☐	☐	☐	☐	☐
Weight gain	☐	☐	☐	☐	☐

18. [If Q9 = "Yes" (has experienced weight gain in the past)] Thinking about the weight you gained because of the medicine(s) you have taken for [schizophrenia / bipolar disorder], how much do you agree with the following statements?

	Not Sure / Not Applicable	Strongly disagree	Disagree	Neither agree nor disagree	Agree	Strongly agree
The weight gain made me feel embarrassed	☐	☐	☐	☐	☐	☐
The weight gain limited my ability to participate in social activities such as going out or seeing friends	☐	☐	☐	☐	☐	☐
The weight gain limited my ability to work	☐	☐	☐	☐	☐	☐
The weight gain interfered with my romantic relationships	☐	☐	☐	☐	☐	☐
The weight gain interfered with my relationships with family/friends	☐	☐	☐	☐	☐	☐
The weight gain affected my ability to fit into my clothes	☐	☐	☐	☐	☐	☐

Questions About Your Mental Health

19. How often do you talk to a doctor or other healthcare provider about your [schizophrenia/bipolar disorder]?

- ☐ I do not regularly see a doctor or healthcare provider
- ☐ Less than once a year
- ☐ Once a year
- ☐ About every 6 months (twice a year)
- ☐ About every 4 months (3 times a year)
- ☐ About every 3 months (4 times a year)
- ☐ More than 4 times a year
- ☐ Don’t know or not sure

20. **[If currently seeing an HCP, Q19 ≠ I do not regularly see a doctor or healthcare provider] Which type of doctor or healthcare provider usually treats your [schizophrenia/bipolar disorder]? *(Please select all that apply.)***

- ☐ General practitioner
- ☐ Psychiatrist
- ☐ Psychologist or counselor
- ☐ Nurse or nurse practitioner
- ☐ Physician assistant
- ☐ Social worker
- ☐ Don't know or not sure [Exclusive]
- ☐ Prefer not to say [Exclusive]

21. **[If currently seeing an HCP, Q19 ≠ I do not regularly see a doctor or healthcare provider] Which type of doctor or healthcare provider usually prescribes your [schizophrenia/bipolar disorder] medications? *(Please select all that apply.)***

- ☐ General practitioner
- ☐ Psychiatrist
- ☐ Nurse or nurse practitioner
- ☐ Physician assistant
- ☐ Don't know or not sure [Exclusive]
- ☐ Prefer not to say [Exclusive]

22. Many people have a hard time remembering to take their medicines. Have you <u>ever</u> skipped or forgotten to take any medicine prescribed for your [schizophrenia/bipolar disorder]?

- ☐ Yes
- ☐ No
- ☐ Don't know or not sure
- ☐ Prefer not to say

23. [If Q22 = Yes] Have you skipped or forgotten to take any medicine prescribed for your [schizophrenia/bipolar disorder] <u>in the past month</u>?

- ☐ Yes
- ☐ No
- ☐ Don't know or not sure
- ☐ Prefer not to say

24. [If Q23 = "Yes"] Which of the following are reasons you skipped or forgot to take your [schizophrenia/bipolar disorder] medicine(s) in the past month? *(Please select all that apply.)*

- ☐ Forgot/too busy
- ☐ Felt well
- ☐ Cost concerns
- ☐ Ran out of medicine or could not easily get a prescription
- ☐ Didn't have my medicine with me (for example, I was travelling or in the hospital)
- ☐ Felt like I needed a break from medicine
- ☐ Worried about gaining weight
- ☐ Medicine or side effects made me feel bad
- ☐ Medicine or side effects interfered with my social, family, or love life
- ☐ Medicine or side effects interfered with my daily activities
- ☐ Something stressful happened in my life
- ☐ Don't know or not sure [Exclusive]
- ☐ Other reason(s)

25. Has a clinician ever diagnosed you with any of the following?
(Please select all that apply.)

- ☐ Attention-deficit/hyperactivity disorder (ADHD) or attention deficit disorder (ADD)
- ☐ Depression
- ☐ Anxiety
- ☐ Posttraumatic stress disorder (PTSD)
- ☐ Obsessive-compulsive disorder
- ☐ Borderline personality disorder
- ☐ Alcohol use disorder
- ☐ Other drug use disorder
- ☐ Heart or circulation problems
- ☐ Kidney problems
- ☐ None of the above [Exclusive]
- ☐ Prefer not to say [Exclusive]

Questions About Your Working Life and Relationships

The following questions ask about the effect of your health problems on your ability to work and perform regular activities. By health problems we mean any physical or emotional problem or symptom.

26. Are you currently employed (working for pay)?

☐ Yes

☐ No

[If Q26 = "No" skip to Q31]

The next questions are about the past seven days, not including today.

27. During the past seven days, how many hours did you miss from work because of your [schizophrenia / bipolar disorder]? Include hours you missed on sick days, times you went in late, left early, etc., because of your health problems. Do not include time you missed to participate in this study.

______ HOURS

28. During the past seven days, how many hours did you miss from work because of any other reason, such as vacation, holidays, time off to participate in this study?

_______ HOURS

29. During the past seven days, how many hours did you actually work?

_______ HOURS [If = 0, skip to Q31]

30. During the past seven days, how much did [schizophrenia / bipolar disorder] affect your productivity while you were working?

Think about days you were limited in the amount or kind of work you could do, days you accomplished less than you would like, or days you could not do your work as carefully as usual. If [schizophrenia / bipolar disorder] affected your work only a little, choose a low number. Choose a high number if health problems affected your work a great deal.

Consider only how much [schizophrenia / bipolar disorder] affected productivity while you were working.

**[schizophrenia / bipolar disorder] had no effect on my work**	0	1	2	3	4	5	6	7	8	9	10	**[schizophrenia / bipolar disorder] completely prevented me from working**

31. During the past seven days, how much did [schizophrenia / bipolar disorder] affect your ability to do your regular daily activities, other than work at a job?

By regular activities, we mean the usual activities you do, such as work around the house, shopping, childcare, exercising, studying, etc. Think about times you were limited in the amount or kind of activities you could do and times you accomplished less than you would like. If [schizophrenia / bipolar disorder] affected your activities only a little, choose a low number. Choose a high number if health problems affected your activities a great deal.

Consider only how much [schizophrenia / bipolar disorder] affected your ability to do your regular daily activities, other than work at a job.

**[Schizophrenia / Bipolar disorder] had no effect on my daily activities**	0	1	2	3	4	5	6	7	8	9	10	**[Schizophrenia / Bipolar disorder] completely prevented me from doing my daily activities**

32. How much do you agree with the following statements, overall?

	Strongly disagree	Disagree	Neither agree nor disagree	Agree	Strongly agree	Not Sure
My schizophrenia/bipolar disorder symptoms have limited my ability to engage in social activities	☐	☐	☐	☐	☐	☐
My schizophrenia/bipolar disorder symptoms have interfered with my romantic relationships	☐	☐	☐	☐	☐	☐
My schizophrenia/bipolar disorder symptoms have interfered with my friendships	☐	☐	☐	☐	☐	☐
My schizophrenia/bipolar disorder symptoms have caused problems with my family	☐	☐	☐	☐	☐	☐

Questions About You

33. What is your height?

______ ft ______in

34. What is your weight?

______ lbs

35. In an average week, how often do you exercise or work out for 30 minutes or more? *(Please select one.)*

- ☐ None
- ☐ 1-3 times
- ☐ 3-6 times
- ☐ Every day or more often
- ☐ Don’t know or not sure
- ☐ Prefer not to say

36. Have you ever tried a diet to help you lose weight? *(Please select one.)*

- ☐ Yes
- ☐ No
- ☐ Don’t know or not sure
- ☐ Prefer not to say

37. Have you ever tried an exercise regimen to help you lose weight? *(Please select one.)*

- ☐ Yes
- ☐ No
- ☐ Don’t know or not sure
- ☐ Prefer not to say

38. What gender identity do you identify with most? ***(Please select one.)***

- ☐ Female
- ☐ Male
- ☐ Gender fluid
- ☐ Nonbinary
- ☐ A gender identity not listed

39. What race(s) or ethnicity(ies) do you consider yourself to be?
(Please s*elect all that apply.*)

- ☐ African American or Black
- ☐ Alaska Native, American Indian, or Native American
- ☐ Asian
- ☐ Hispanic, Latin American, or Latinx
- ☐ Middle Eastern or North African
- ☐ Native Hawaiian or Pacific Islander
- ☐ White
- ☐ A race or ethnicity not listed

40. What is your marital status? *(Please select one.)*

- ☐ Single/never married
- ☐ Married/living as married/civil partnership
- ☐ Divorced or separated
- ☐ Widowed/surviving partner
- ☐ Other

41. What is the highest level of education you have completed? *(Please select one.)*

- ☐ Less than high school
- ☐ Some high school
- ☐ High school or equivalent (for example, GED)
- ☐ Some college but no degree
- ☐ Associate’s degree (2-year college degree)
- ☐ 4-year college degree (for example, BA, BS)
- ☐ Some graduate school but no degree
- ☐ Graduate or professional degree (for example, MBA, MS, MD, PhD)

42. Which of the following best describes your current employment? (*Please select all that apply.*)

- ☐ Employed full time
- ☐ Employed part time
- ☐ Employed on a temporary or seasonal contract
- ☐ Self-employed or freelancer
- ☐ Homemaker
- ☐ Student
- ☐ Retired
- ☐ Off work/unable to work because of disability or other health condition
- ☐ Unemployed and looking for work
- ☐ Unemployed and not looking for work
- ☐ Prefer not to say [Exclusive]

A Few Questions About COVID-19

On a scale from 0 to 5 where 0 is strongly disagree and 5 is strongly agree, how much to you agree with the following two statements

43. **[If currently seeing an HCP, Q19 ≠ I do not regularly see a doctor or healthcare provider] Since the start of COVID-19, discussing [schizophrenia/bipolar disorder] symptoms and treatment with my healthcare provider has become easier**

Strongly disagree	0	1	2	3	4	5	Strongly Agree

☐ Not applicable, I was diagnosed with [schizophrenia/bipolar disorder] after the start of the pandemic

44. **Overall, COVID-19 has changed my life, in general, in a negative way**

Strongly disagree	0	1	2	3	4	5	Strongly agree

45. Since the COVID-19 pandemic started, how have you been receiving most of your mental healthcare for your [schizophrenia/bipolar disorder]? (*Please select all that apply.*)

- ☐ Face-to-face appointments
- ☐ Telephone appointments
- ☐ Online, video, and/or app-based appointments
- ☐ I do not have regular appointments for my mental health [Exclusive]
- ☐ Don't know or not sure [Exclusive]

46. [If diagnosed before 2019/ If Q43 ≠ Not Applicable] How did you receive most of your mental healthcare for your [schizophrenia/bipolar disorder] before the COVID-19 pandemic? *(Please select all that apply.)*

- ☐ Face-to-face appointments
- ☐ Telephone appointments
- ☐ Online, video, and/or app-based appointments
- ☐ I did not receive regular appointments for my mental health before the pandemic [Exclusive]
- ☐ I was diagnosed after the start of the pandemic [Exclusive]
- ☐ Don't know or not sure [Exclusive]

Thank you for taking the time to complete this survey.
