# Supplementary material for: Preferences for attributes of oral antipsychotic treatments: results from a discrete-choice experiment in respondents with schizophrenia or bipolar I disorder
Source: BMC Psychiatry. 2024 Sep 10;24:605. doi: 10.1186/s12888-024-06034-1 (PMC11389064; doi:10.1186/s12888-024-06034-1)
Supplement: Supplementary file 4 — Additional file 4: Analysis of preference data [file 12888_2024_6034_MOESM4_ESM.pdf]

Additional File 4. Analysis of preference data

Data obtained from DCE surveys can be analyzed with a series of models that are defined as mixed-logit models [1, 2]. Preference data were analyzed using random-parameter logit (RPL) models [3]. In the utility specification considered in the analysis, all parameters were effects-coded (such that the parameter for the omitted category was the negative sum of the included categories, and the 95% CI was retrieved using the delta method) instead of dummy-coded. Thus, the mean effect for each attribute was normalized at 0 rather than having a 0 value corresponding to the set of all the omitted categories. In the RPL model specification, all parameters were assumed to be normally distributed and independent. Model estimates were used to compute conditional relative importance values and the maximum acceptable weight gain (MAWG) and maximum acceptable risk (MAR) [4].

The MAWG was defined as the negative ratio between the marginal utility of a specific improvement in an attribute and the marginal disutility of 1 lb of weight gain:

$$\text{MAWG} = -\frac{\Delta\beta X}{\Delta\alpha W}$$

where $\Delta\beta X$ is the utility gained by increasing antipsychotic efficacy and $\Delta\alpha W$ is the disutility of increases in weight. Given the hypothetic preference coefficient estimates (β),

- utility of going from “a little” to “some” disease improvement, 1.5, and
- disutility of an increase in weight gain from 0 to 4 lb, −0.5,

$$\text{MAWG} = -\frac{1.5}{\frac{-0.5}{4-0}} = 12\ \text{lb}$$

This ratio provides the average weight gain increase that yields a level of disutility equal to the utility generated by improving treatment efficacy 1 level, from “a little” to “some.” However, the MAWG is greater than 4 lb (the difference between the first 2 levels of the risk considered for the MAWG), so the total MAWG must consider the change in the slope of the disutility of increases in weight gain. Given the following hypothetical preference coefficient estimate,

- disutility of an increase in weight gain from 4 to 7 lb, −0.8,

$$\text{MAWG} = -\frac{1.5 - 0.5}{\frac{-0.8}{7-4}} + 4 = 7.75\ \text{lb}$$

Again, the total MAWG must consider the change in the slope of the disutility of increases in weight gain, from 7 to 11 lb. Given the following hypothetical preference coefficient estimate,

- disutility of an increase in weight gain from 7 to 11 lb, −0.4,

$$\text{MAWG} = -\frac{1.5 - 0.5 - 0.8}{\frac{-0.4}{11-7}} + 7 = 9.0\ \text{lb}$$

Likewise, the MAR was defined as the negative of the ratio between the marginal utility of a specific improvement in an attribute and the marginal disutility of each risk,

$$\text{MAR} = -\frac{\Delta\beta X}{\Delta\alpha R}$$

where $\Delta\beta X$ is the utility gained by increasing antipsychotic efficacy and $\Delta\alpha R$ is the disutility of increases in risk. Given the following hypothetical preference coefficient estimates,

- utility of going from “a little” to “some” disease improvement, 1.5, and
- disutility of an increase in the risk of sedation from 0% to 10%, −0.5,

$$\text{MAR} = -\frac{1.5}{\frac{-0.5}{10-0}} = 30\%$$

This ratio provides the average percent increase in treatment-related risk that yields a level of disutility equal to the utility generated by improving treatment efficacy 1 level, from “a little” to “some.” However, the MAR is greater than 10% (the difference between the first 2 levels of the risk considered for the MAR), so the total MAR must consider the change in slope of the disutility of increases in risk. Given the following hypothetical preference coefficient estimate,

- disutility of an increase in the risk of sedation from 10% to 25%, −0.8,

$$\text{MAR} = -\frac{1.5 - 0.5}{\frac{-0.8}{25 - 10}} + 10 = 28.75\%$$

The MAR is greater than 25%. Instead of making the strong assumption that the disutility of each unit increase in risk remains constant beyond 25%, the MAR was considered >25%.

REFERENCES

1. McFadden D, Train K. Mixed MNL models for discrete response. J Appl Econ (Chichester Engl). 2000;15:447-70. https://doi.org/10.1002/1099-1255(200009/10)15:5<447::AID-JAE570>3.0.CO;2-1.

2. Hauber AB, González JM, Groothuis-Oudshoorn CG, Prior T, Marshall DA, Cunningham C, MJ IJ, Bridges JF. Statistical methods for the analysis of discrete choice experiments: a report of the ISPOR Conjoint Analysis Good Research Practices Task Force. Value Health. 2016;19:300-15. 10.1016/j.jval.2016.04.004.
3. Vass C, Boeri M, Karim S, Marshall D, Craig B, Ho KA, Mott D, Ngorsuraches S, Badawy SM, Mühlbacher A, et al. Accounting for preference heterogeneity in discrete-choice experiments: an ISPOR Special Interest Group report. Value Health. 2022;25:685-94. 10.1016/j.jval.2022.01.012.
4. Gonzalez JM, Boeri M. The impact of the risk functional form assumptions on maximum acceptable risk measures. The patient. 2021;14:827-36. 10.1007/s40271-021-00518-y.
